# Commentary: Measuring Depression in a Non-Western War-Affected Displaced Population: Measurement Equivalence of the Beck Depression Inventory

**DOI:** 10.3389/fpsyg.2018.00566

**Published:** 2018-04-18

**Authors:** Miyuru Chandradasa, Layani Champika

**Affiliations:** ^1^Department of Psychiatry, Faculty of Medicine, University of Kelaniya, Ragama, Sri Lanka; ^2^School of Rural Health, Faculty of Medicine Nursing and Health Sciences, Monash University, Melbourne, VIC, Australia; ^3^Consultation Liaison Psychiatry, Latrobe Regional Hospital, Traralgon, VIC, Australia

**Keywords:** psychometrics, mental health, depression, Sri Lanka, war exposure, transcultural studies

Frontiers in Psychology has published the article titled “Measuring Depression in a Non-Western War-Affected Displaced Population: Measurement Equivalence of the Beck Depression Inventory” by Jayawickreme et al. recently (Jayawickreme et al., 2017). As Sri Lankan mental health professionals, we believe this is an important publication that allows the use of psychometrics in non-Western war-affected populations that have different linguistic and cultural contexts to the Western world. Firstly, we would like to highlight the importance of validating culturally relevant psychometric tools to resource-limited settings in the developing world. Secondly, we emphasize the need to focus on validating freely available tools by researchers so that these studies would be more useful on the ground level.

Sri Lanka is a South Asian middle-income nation with a literacy rate close to 93%. A three-decade-old armed conflict in the country ended in 2009. Since the end of the war, the country's gross domestic product has doubled. In relevance to the medical workforce, there were 0.726 doctors per 1,000 population in 2010, which would have increased over the last few years (World Bank, 2018). According to the data provided by the World Health Organization in 2011, there were 0.29 psychiatrists per 100,000 population (World Health Organization, 2011). According to the Postgraduate Institute of Medicine in Sri Lanka, 121 general adult psychiatrists, four child and adolescent psychiatrists and two forensic psychiatrists have obtained board certification from 1983 to 2017 (Postgraduate Institute of Medicine, 2017). However, some of these specialists left the country during times of war. A significant number of psychiatrists who left the country for advanced training in the United Kingdom and Australia have returned to the country since the end of the war (Chandradasa and Kuruppuarachchi, 2017). By 2015, there were 63 psychiatrists working for the Ministry of Health in the public health sector of Sri Lanka (De Silva, 2017). By adding the number of psychiatrists attached to Universities and private sector, it could be assumed that Sri Lanka's current psychiatrist per population ratio is around 0.5 per 100,000 population. In whatever the method of estimation, it is evident that the number of psychiatrists per population is far below the figures for more affluent countries of the world. Clinical psychologists are not routinely recruited to the public health system and there is a limited number of other allied health professionals (World Health Organization, 2011).

Due to the shortage of mental health professionals in the country, the provision of psychiatric care for the population has been difficult over the past few decades (Chandradasa and Kuruppuarachchi, 2017). The estimated mental health gap for the conflict-affected regions of the country is even higher (Siriwardhana et al., 2013). Jayawickreme et al. conducted the study among Tamil speaking females affected by the war in districts of Jaffna, Batticaloa, Trincomalee, and Vavuniya where the availability of mental health services is far limited compared to the urban regions of the country. Therefore, the availability of psychometric instruments to screen for psychological distress in traumatized community groups is important to detect the affected individuals early and refer to obtain appropriate management.

A group of researchers from the University of Kelaniya Sri Lanka has validated the Beck Depression Inventory for the Sinhalese population in 2015 (Rodrigo et al., 2015). The Tamil version has been validated and used in previous studies in the country (Jayawickreme et al., 2012). The Beck Depression Inventory has been in use for many decades and it has been found to be a valid psychometric instrument in many different settings worldwide (Beck et al., 1996). However, the scale is not freely available for use in clinical practice (Pearson Education, 2017). The price of the manual and a set of forms would present a substantial cost by Sri Lankan standards and a community screening of 100 people would amount to more than one month's salary of a Sri Lankan psychiatrist. The health authorities do not fund for psychometric instruments routinely and they are usually purchased by individual researchers or clinicians.

There are culturally validated psychometric instruments to detect depressive symptoms in the public domain. For example, the Centre for Epidemiologic Studies Depression Scale (CES-D) is validated to Sinhalese and Tamil languages and has been used in the country (Radloff, 1977). This instrument is available in 10 and 20 item versions and is found to be an easy to use, self-report psychometric instrument for depressive symptoms. The CES-D has been used successfully in severely trauma-affected populations in the past such as Rwandan genocide survivors (Lacasse et al., 2014) and prisoners of World War II and Korean War (Engdahl et al., 1991).

By this publication, Jayawickreme et al have contributed immensely to improve the knowledge on the use of culturally valid psychometric instruments for war-affected communities in Sri Lanka. It would be highly appropriate if further research is done to validate instruments that are freely available to be used in ground level clinical services for these psychologically vulnerable populations.

## Author contributions

MC: Design, concept and writing of the manuscript; LC: Literature review, writing of the manuscript.

### Conflict of interest statement

The authors declare that the research was conducted in the absence of any commercial or financial relationships that could be construed as a potential conflict of interest.
